# What Is There in Seeds? Vertically Transmitted Endophytic Resources for Sustainable Improvement in Plant Growth

**DOI:** 10.3389/fpls.2018.00024

**Published:** 2018-01-23

**Authors:** Raheem Shahzad, Abdul L. Khan, Saqib Bilal, Sajjad Asaf, In-Jung Lee

**Affiliations:** ^1^School of Applied Biosciences, Kyungpook National University, Daegu, South Korea; ^2^Chair of Oman's Medicinal Plants and Marine Natural Products, University of Nizwa, Nizwa, Oman

**Keywords:** seed endophytes, vertical transmission, metabolite production, plant growth, stress mitigation

## Abstract

Phytobeneficial microbes, particularly endophytes, such as fungi and bacteria, are concomitant partners of plants throughout its developmental stages, including seed germination, root and stem growth, and fruiting. Endophytic microbes have been identified in plants that grow in a wide array of habitats; however, seed-borne endophytic microbes have not been fully explored yet. Seed-borne endophytes are of great interest because of their vertical transmission; their potential to produce various phytohormones, enzymes, antimicrobial compounds, and other secondary metabolites; and improve plant biomass and yield under biotic and abiotic stresses. This review addresses the current knowledge on endophytes, their ability to produce metabolites, and their influence on plant growth and stress mitigation.

## Background

Soil hosts a diverse array of microbes, such as bacteria, fungi, yeasts, and protozoa. These microbes often exist in mutualistic interactions; some are also found in mutual relationships with plants (Farrer and Suding, 2016; Vejan et al., 2016; Lladó and Baldrian, 2017). These plant–microbe associations have been the focus of comprehensive study, given their potential as ecologically sound alternatives for promoting crop growth and development. It is clear that microorganisms are able to enhance plant growth and defenses, and that plants have the ability to select a microbiome in order to retain valuable colonizers, including those living within their tissue (Hardoim et al., 2012; Marasco et al., 2012; Rashid et al., 2012). Within this context, seed microbiota are ecologically interesting in that they represent not only an endpoint for the community assembly in the seed, but also a starting point for community assembly in the new seedling.

The present review concentrates on underexplored endophytes, such as seed-borne bacterial and fungal endophytes. The review considers their role in enhancing crop efficiency, the nature of vertical transmission and secondary metabolite production, their below-ground function, and the above-ground response.

## Endophytic microbes: role and reproduction

Recent evaluations suggest that over 300,000 plant species are found worldwide, and that every plant carries at least one endophyte (Smith et al., 2008). Indeed, endophytic microbes have been found in every plant species examined to date; Partida-Martínez and Heil (2011) report that a plant without endophytes could only occur infrequently. It can be assumed that plants deprived of endophytes would be more vulnerable to environmental stress and pathogenic attacks (Khan et al., 2015; Leitão and Enguita, 2016; Suman et al., 2016; Brader et al., 2017). Endophytic microorganisms (bacteria or fungi) are a key class of plant symbionts that live inside plant tissues without inducing any disease symptoms (Brader et al., 2017), and which are associated with the plant throughout its life history, from seed germination to fruit development. Endophytes are found in the roots (rhizosphere), leaves (phylloplane), stems (laimosphere and caulosphere), fruits (carposphere), seeds (spermosphere), and flowers (anthosphere), as described by many scientists (Clay and Holah, 1999; Lindow and Brandl, 2003; Saikkonen et al., 2004; Shahzad et al., 2016; Brader et al., 2017). The relationship between endophytes and plants is unique in the ability of the former to provide alternative sources of biologically active metabolites, such as enzymes, biofunctional chemicals, phytohormones, nutrients, and minerals, and to facilitate the distribution or production of these resources which contributed in the elimination of various stresses (Schulz et al., 2002; Khan et al., 2012; Kong and Glick, 2017; Nelson, 2017). In return, the host plant provides a protective sanctuary for the microbes within the plant tissues, in which they can grow and reproduce, but without compromising the plant's own growth resources (Khan et al., 2015).

## Why are endophytes in seeds important?

Seeds play an important role in the life cycle of spermatophytes; they have the ability to exist in a torpid state for a considerable length of time until growth conditions are suitable, and then develop into a new plant (Nelson, 2004; Geisen et al., 2017). It is probable that seeds benefit from seed-borne bacterial and fungal endophytes, which are thought to promote seed conservation and facilitate seed germination in soil (Chee-Sanford et al., 2006; Rodríguez et al., 2017; Shearin et al., 2017). Seed-borne endophytes are of particular importance because they are passed between successive plant generations via vertical transmission, thus ensuring their presence in the next generation of seedlings (Cope-Selby et al., 2017; Shade et al., 2017). This process of vertical transmission results in a weakening of microbial pathogenic strength in order to support plant growth and development. This mutualism supports and enhances plant survival and microbial proliferation (Rudgers et al., 2009). Moreover, alongside their vital role in plant growth and defense, these seed-borne bacterial and fungal endophytes benefit the host plants through providing their offspring with valuable endosymbionts (Shade et al., 2017).

## Biodiversity trove in seeds

The internal environment of a seed changes during maturation, which consequently affects the seed endophytic community (Mano et al., 2006). The ability to reside in a seed and adapt to severe environmental conditions are special characteristics of seed endophytes that are rarely found in endophytes isolated from roots, shoots, or other plant tissues. Seed endophytes have the ability to form endospores, thus providing protection from changing conditions inside the seed (Mano et al., 2006; Compant et al., 2011; Kane, 2011). They also maintain other features, such as cell motility and phytase activity, in order to be able to migrate freely inside the plant and enter the seeds before they harden. There have however been relatively few studies examining biodiversity in seed-borne endophytes.

## Seed-borne bacterial endophytes

The various seed-borne bacterial endophytes found in plant tissues utilize either direct or indirect mechanisms to improve plant growth and development, and enhance plant tolerance to biotic and abiotic stresses (Santoyo et al., 2016; Shahzad et al., 2017a,b). They facilitate plant development by activating supplements in the soil, delivering plant hormones, controlling or hindering phytopathogens to defend the plant, enhancing soil structure, and bioremediating contaminated soils by sequestering dangerous metals and degrading xenobiotic mixes (Maehara et al., 2016; Sülü et al., 2016). Seed-borne bacterial endophytes also participate in modulating endogenous phytohormones (Shahzad et al., 2016). In addition, some plant growth-promoting bacterial endophytes can lower ethylene levels by synthesizing a catalyst, ACC deaminase (1-aminocyclopropane-1-carboxylate), of an ethylene precursor in higher plants (Mano et al., 2006; Sziderics et al., 2007; Doty et al., 2009; Glick, 2012; Luo et al., 2012; Rashid et al., 2012; Coutinho et al., 2014; Pandya et al., 2015; Saini et al., 2015). Although very few studies have examined the biodiversity of seed-borne bacterial endophytes, seeds from numerous plant species have been shown to contain diverse communities of bacterial endophytes (Table 1).

**Table 1:** Endophytic microbes isolated and characterized from the seeds of different plants.

**Host**	**Endophytic microbes**	**Function**	**References**
**BACTERIA**			
*Oryza sativa*	*Paenibacillus polymyxa*	Glucanase production, anti-phytopathogenic microbe	Liu et al., 2017
*Cucumis melo*	*Proteobacteria, Frimicutes, Actinobacteria*	–	Glassner et al., 2017
*Oryza sativa*	*Micrococcus yunnanensis, Micrococcus luteus, Enterobacter soli, Leclercia adecarboxylata, Pantoea dispersa, Staphylococcus epidermidis*	IAA production, plant growth promotion	Shahzad et al., 2017c
*Oryza sativa*	*Enterobacter asburiae, Pantoea dispersa, Pseudomonas putida*	IAA production, phosphate-solubilizing, antifungal, plant growth promotion	Verma et al., 2017
*Phragimates australis*	*P. fluorescens, Psedomonas* sp., *Pantoea* sp., *Enterobacter* sp.	Phosphorus-solubilizing, protease production, anti-fungal, plant growth promotion	White et al., 2017
*Triticum aestivum*	*Panibacillus* sp., *Pantoea* sp., *Bacillus* sp.	IAA production, antifungal, siderophore production, phosphate-solubilizing, plant growth promotion	Díaz Herrera et al., 2016
*Tylosema esculentum*	*Massilia, Kosakonia, Pseudorhodoferax, Caulobacter, Pantoea, Sphingomonas, Burkholderia, Methylobacterium, Bacillus* sp., *Curtobacterium, Microbacterium, Mucilaginibacter, Chitinophaga*	Plant growth promotion, phytohormone and metabolite production	Chimwamurombe et al., 2016
*Oryza sativa*	*Bacillus amyloliquefaciens*	Phytohormone production, growth promotion	Shahzad et al., 2016
*Lycopersicum esculentum*	*Bacillus subtilis*	Plant growth promotion, phytohormone and metabolite production	Xu et al., 2014
*Zea mays*	*Undibacterium, Sphingomonas, Acinetobacter, Burkholderia, Pantoea, Limnobacter, Burkholderia, Pantoea, Staphylococcus, Serratia, Cronobacter, Enterobacter, Escherichia, Acinetobacter*	–	Liu et al., 2013
*Arachis hypogaea*	*B. thuringiensis, B. cereus, B. amyloliquefaciens, B. megaterium, B. subtilis, Bacillus* sp., *Paenibacillus* sp., *Pseudomonas* sp., *B. thioparans, Cyanobacterium*	Antifungal	Sobolev et al., 2013
*Phaseolus vulgaris*	*Bacillus massilensis, Bacillus* sp. *Bacillus pumilus, Bacillus flexus, Bacillus korlensis, Bacillus silvestris, Paenibacillus, Enterococcus, Staphylococcus, Arthrobacter, Kocuria, Micrococcus, Brachybacterium, Methylobacterium, Paracoccus, Acinetobacter*	–	Rosenblueth et al., 2012
*Triticum aestivum; Elymus trachycaulus; Agropyron fragile*	*Actinobacteria, Firmicutes, Gammaproteobacteria*	–	Ringelberg et al., 2012
*Oryza sativa*	*Pseudomonas protegens, Pseudomonas* sp., *Stenotrophomonas maltophilia, Uncultured Stenotrophomonas clone, Ochrobactrum tritici, Ochrobactrum* sp., *Ochrobactrum grignonense Sphingomonas yanoikuyae, Flavobacterium johnsoniae, Flavobacterium* sp., *Paenibacillus humicus, Paenibacillus* sp. *Agromyces mediolanus, Curtobacterium citreum, Curtobacterium* sp., *Curtobacterium herbarum, Frigoribacterium faeni, Microbacterium oleivorans, Microbacterium* sp., *Mycobacterium abscessus Plantibacter flavus*	Plant growth promotion, mitigating biotic and abiotic stress	Hardoim et al., 2012
*Zea mays*	*Bacillus* sp, *Methylobacterium, Tukamurella, Alcaligenes, Erwinia, Microbacterium, Rhodococcus*	–	Rosenblueth et al., 2012
*Cucurbita pepo*	*Bacillus sp., Pseudomonas chlororaphis, Lysobacter gummosus, P. chlororaphis, Paenibacillus polymyxa, Serratia plymuthica*	Antifungal	Fürnkranz et al., 2012
*Vitis vinifera*	*Bacillus altitudinis, Bacillus simplex, Bacillus thuringiensis, Paenibacillus amylolyticus, Staphylococcus aureus* subsp. a*ureus*	Tissue colonization	Compant et al., 2011
*Fraxinus*	*Pantoea agglomerans, Staphylococcus succinus, Aerococcus viridans*	Antibiotic production	Donnarumma et al., 2011
*Oryza sativa*	*Pantoea agglomerans, Acinetobacter* sp., *Curtobacterium citreum, Microbacterium* sp., *Pantoea ananatis, Pseudomonas* sp., *Paenibacillus* sp., *Pantoea* sp., *Staphylococcus cohnii, Curtobacterium citreum, Microbacterium* sp., *Sphingomonas* sp., *Rhizobium larrymoorei, Curtobacterium* sp., *Sphingomonas* sp.	Phytohormone and metabolite production, phosphate-solubilizing, antifungal, plant growth promotion	Ruiza et al., 2011
*Glycine max*	*Acinetobacter, Bacillus, Enterococcus, Nocardioides, Paracoccus, Phyllobacterium, Sphingomonas*	Phytate-solubilizing	López-López et al., 2010
*Nicotiana tabacum*	*Enterobacter* sp., *Xanthomonadaceae, Pseudomonas* sp., *Enterobacter* sp., *Pseudomonas fulva, Sanguibacter* sp., *Stenotrophomonas* sp., *Clostridium aminovalericum, Stenotrophomonas* sp., Sanguibacter sp.	Mitigating metal toxicity, promote plant growth	Mastretta et al., 2009
*Oryza sativa*	*Bacillus pumilus, Kocuria palustris, Pantoea ananatis, Methylobacterium radiotolerans, Methylobacterium fujisawaense*	Enzyme production, osmotic stress tolerance	Kaga et al., 2009
*Eucalyptus*	*Bacillus* sp., *Enterococcus* sp., *Paenibacillus* sp., *Methylobacterium* sp.	Growth promotion	Ferreira et al., 2008
*Zea mays*	*Pantoea* sp., *Microbacterium* sp., *Frigoribacterium* sp., *Bacillus* sp., *Paenibacillus* sp., *Sphingomonas* sp.	Antifungal	Rijavec et al., 2007
*Oryza sativa*	*Xanthomonas translucens, Pantoea ananatis, Methylobacterium aquaticum, Sphingomonas melonis, Sphingomonas yabuuchiae, Bacillus subtilis, Bacillus pumilus, Micrococcus luteus, Acidovorax* sp., *Curtobacterium flaccumfaciens, Paenibacillus amylolyticus, Xanthomonas translucens*	Enzyme production, osmotic stress tolerance	Mano et al., 2006
*Coffea Arabica*	*Bacillus* sp., *Burkholderia cepacia—GC subgroup B, Burkholderia gladioli GC subgroup A, Burkholderia gladioli—GC subgroup B, Clavibacter michiganense insidiosum, Curtobacterium flaccumfaciens-flaccumfaciens, Curtobacterium flaccumfaciens-poinsettiae, Escherichia vulneris, Micrococcus* sp., *Pantoea agglomerans, Pseudomonas putida* biotype A, *Pseudomonas putida* biotype B, *Stenotrophomonas* sp., *Stenotrophomonas maltophilia, Yersinia frederiksenii*	–	Vega et al., 2005
*Fragaria*	*Pseudomonas fluorescens, Pseudomonas* sp.	–	Kukkurainen et al., 2005
*Glycine max*	*Agrobacterium radiobacter, Aeromonas* sp., *Bacillus* spp., *Chryseomonas luteola, Flavimonas oryzihabitans, Sphingomonas paucimobilis*	Seedling growth, root colonization	Oehrle et al., 2000
**FUNGI**			
Invasive *Phragmites*	*Alternaria* sp., *Phoma* sp., *Penicillium corylophilum*	Improved seed germination and seedling growth	Shearin et al., 2017
*Dendrobium friedericksianum*	*Fusarium* sp., *Beauveria* sp., *Tulasnella violea, T*. *violea, Epulorhiza* sp., *Trichosporiella multisporum*	Growth promotion	Khamchatra et al., 2016
*Cinchona ledgeriana*	*Diaporthe* sp.	Alkaloid production	Maehara et al., 2016
*Toona sinensis* Roem	*Cladosporium* sp.	Antioxidant potential	Rahmawati et al., 2016
*Lolium perenne*	*Neotyphodium* sp.	–	Wiewióra et al., 2015
*Schedonorus phoenix*	*Epicholë ceonophiala*	Improved resistance against herbivores and environmental stresses	Young et al., 2013
*Dactylis glomerata*	*Epichloë typhina*	Improved host plant growth and photosynthesis	Rozpadek et al., 2015
*Centaurea cyanus;* *Papaver rhoeas;* *Senecio vulgaris;* *Centaurea nigra;* *Plantago lanceolata;* *Rumex acestosa*	*Acremonium strictum, Alternaria alternate, Aspergillus niger, Aureobasidium pullulans, Botrytis cinerea, Chaetomium cochliodes, Clodosporium cladospriodes, Cladosporium oxysporum, Cladosporium sphaerospermum, Colletotrichum dematium, Epicoccum nigrum, Fusarium avenaceum, Fusarium equiseti*, *Fusarium merismoides, Fusarium tricinctum, Fusarium* sp. A*, Geotrichum candidum, Mucor hiemalis, Penicillium* sp A*, Penicillium* sp. B*, Phialophora verrucosa, Rhabdospora coricea, Sterile* sp. A*, Sterile* sp. B		Hodgson et al., 2014
*Laelia speciosa*	*Helotiales* sp.	–	Ávila-Díaz et al., 2013
*Ipomoea carnea*	*Collelotrichum* sp., *Fusarium* sp.	Antimicrobial	Tayung et al., 2012
*Swietenia macrophylla* King	–	α –Glucosidase inhibition	Ramdanis et al., 2012
*Festuca arundinacea*	*Neotyphodium oenophialum*	Ergovaline and loline alkaloid production and improved protection against herbivores	Pennell et al., 2010
Lolium perenne	*Epichloë festucae* var. *lolii*	Improved drought tolerance	Kane, 2011

## Seed-borne fungal endophytes

Fungal endophytes are found in all types of plant tissue, and have been shown to improve growth, enhance plant defense systems, and mitigate both biotic and abiotic stress (Khan et al., 2015). Endophytic fungi reveal a broad variation in their mode of transmission from one host to another, and stringent vertical transmission from one generation to the next (Shearin et al., 2017; Vujanovic and Germida, 2017). Many fungi are seed-borne, and very recent studies report that fungal seed microbiomes may be greatly influenced by local conditions and non-host genotypes (Klaedtke et al., 2016). The well-studied seed-borne fungal endophytes belonging to the genus *Epichlöe* are mostly reported to assist their host plants in growth promotion and stress mitigation, either directly or indirectly (Kauppinen et al., 2016; Gundel et al., 2017). However, although research has focused on this group of fungi, there are numerous other seed-associated fungi, including ascomycetes, basidiomycetes, parasites, and yeasts (Abe et al., 2015).

In a stringent vertical transmission process, the seeds produced by separate plants are infected with at least one endophyte, but this is not the case for processes involving seed-borne endophytic fungi, in which every seed produced by a single plant may be individually infected with a different fungus. Barret et al. (2015) have determined that the seeds of plants in Brassicaceae were overwhelmingly inhabited by ascomycetes in the classes Dothideomycetes, Eurotiomycetes, Leotiomycetes, and Sordariomycetes, and from the Basidiomyceta. Dothideomycetes is the largest known class of filamentous ascomycetes, and comprises the genera *Alternaria, Aureobasidium, Cladosporium, Epicoccum, Phaeosphaeria, Phoma, Pyrenophora*, and *Stagonospora*. The other ascomycetes classes include typical endophytic genera, such as *Chaetomium, Fusarium, Microdochium, Stemphylium*, and *Xylaria* (Barret et al., 2015). Different seeds bear a variety of fungal endophytes (Table 1).

## Mechanisms of action of seed-borne endophytes

**Figure 1:**
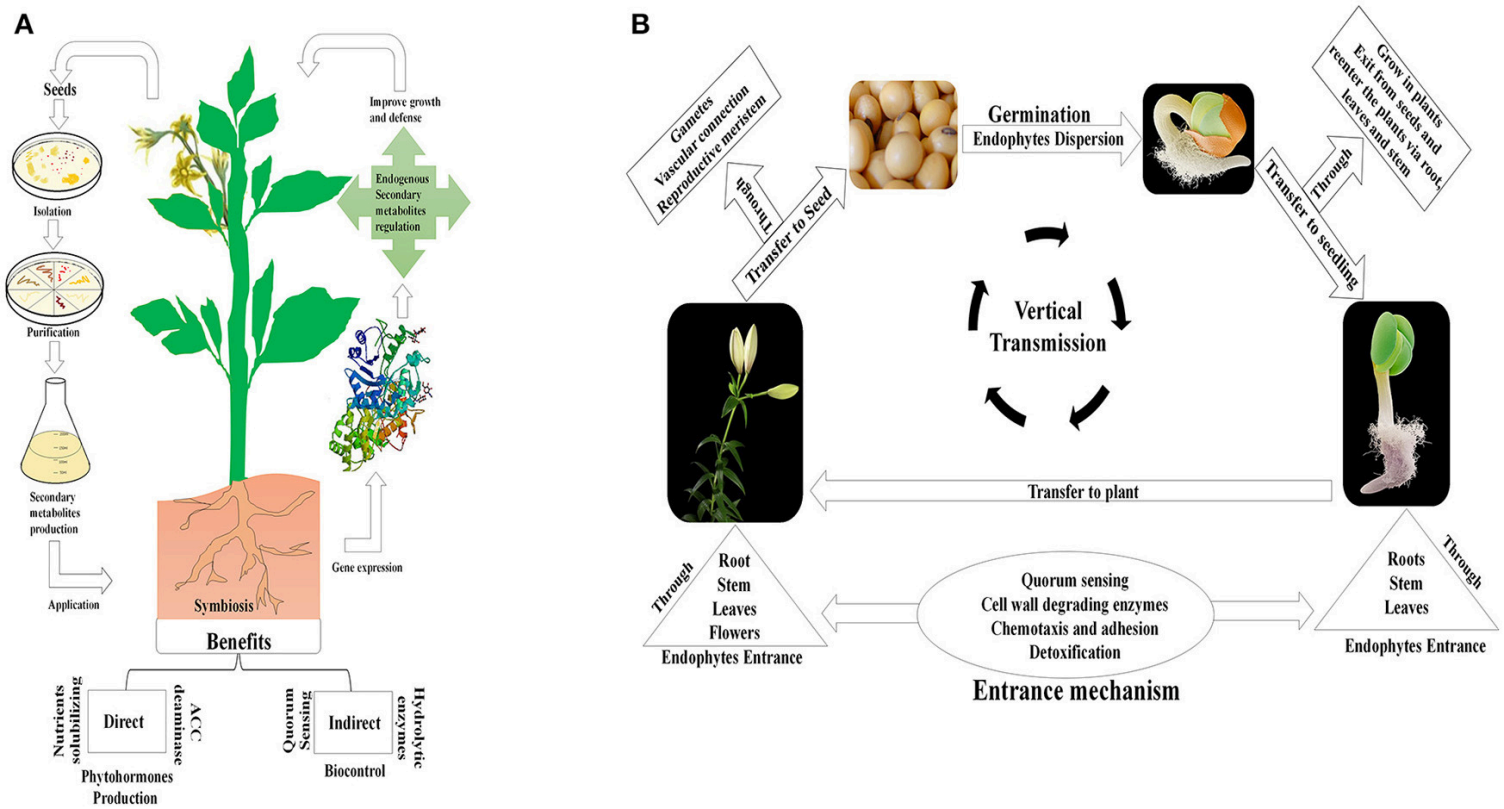
Conceptual view of mechanisms of action and vertical transmission of seed endophytic microbiota. **(A)** The schematic presentation shows the isolation of seed-borne endophytic microbes, and their application in promoting plant growth and stress resistance. **(B)** A holistic view of the vertical transmission of seed-borne endophytes. This suggests that endophytes are found in seed embryos and grow into the emerging leaf upon germination; the endophytes then migrate into the stem and seed head of reproductive plants via various pathways.

The assorted metabolic qualities of seed-borne bacterial and fungal endophytes are dependent on local conditions, and are used to facilitate the host plant's advancement. This further strengthens the benefits conferred on the host plant, improving its fitness over other plants; this can in turn influence the entire environment (Klironomos, 2002; Khan et al., 2015; Figure 1A).

## Mode for vertical transmission of seed-borne endophytes

Seed endophytes must possess efficient motility, and use different means to enter and become established in the seed tissue. They are transmitted either through vascular connections between the vegetative plant parts, the seed and from parental plants into the seed endosperm, or through transgenerational transfer via vertical transmission (Hodgson et al., 2014). Three main transmission pathways have been reported for the transmission of seed born-microbes: (i) via non-vascular or xylem tissues in the maternal plant; (ii) through floral pathways, via the stigma of maternal plants; and (iii) by an exogenous pathway whereby seeds are contaminated from the external environment (Maude, 1996). The relative importance to plants of the horizontal and vertical transmission of microbes remains unclear (Vandenkoornhuyse et al., 2015). However, vertical transmission is reported probably to be a widespread phenomenon in ubiquitous endophytes (Hodgson et al., 2014). This mode of transmission is fascinating in terms of its ability to fortify a plant with an established beneficial endophytic community which can be passed, together with its beneficial traits, to the plant's offspring (Ferreira et al., 2008). Conservation of vertically transmitted endophytes indicates an evolved form of mutualism or benign parasitism in the relationship with the host plant (Johnston-Monje and Raizada, 2011; Figure 1B).

Vertical transmission of seed-borne bacterial and fungal endophytes has been detected in various plant species. By isolating *Bacillus* spp. and *Microbacterium* spp. from switch grass seeds harvested in 1 year and from the plants grown from these seeds the following year, showed that the same microbial species occurred in multiple (Gagne-Bourgue et al., 2013). In addition, Ringelberg et al. (2012) isolated the same endophytic bacterial genera from both seeds and mature plant tissues in wheatgrass, therefore suggesting that the seeds are a key source of transmitting mature wheatgrass endophytes to the next generation. Furthermore, although fungal endophytes were originally thought to be horizontally transmitted, their vertical transmission in various plant species has been reported (Ngugi and Scherm, 2006; Hodgson et al., 2014; Wiewióra et al., 2015). Some studies indeed report that the rate of vertical transmission for many fungal endophytes is greater than 90% (Ngugi and Scherm, 2006).

## Metagenome analysis

Seeds are not merely the carriers of a plant's hereditary information, but also both reservoirs for plant microbiota and vehicles for their vertical transmission (Baker and Smith, 1966; Nelson, 2004). The role of seed-associated microbes is of significance to plant growth and development because these microbial communities may secrete important phytohormones, such as cytokinins, that break seed dormancy (Goggin et al., 2015) and inhibit microbial invasions (Bacilio-Jiménez et al., 2001). The recent rapid progress in high-throughput DNA sequencing technology has enabled a far wider exploration of microbes in the rhizosphere, endosphere, and phyllosphere of important crops and model plant species, revealing the distinctive microbial community structures, which are dependent on the plant parts they inhabit and environmental conditions (Redford and Fierer, 2009; Bulgarelli et al., 2012; Bodenhausen et al., 2013; Shakya et al., 2013; Lebeis, 2014). Furthermore, multi-omics techniques, such as whole genome and metagenomic analyses, have significantly improved our understanding of the role of the plant microbiome (Bai et al., 2015; Bulgarelli et al., 2015).

Extensive attention has been given to the construction and role of microbial communities associated with the phyllosphere and rhizosphere. However, we have a comparatively poor understanding of the microbiota inhabiting other niches, such as the reproductive organs and seeds. Seeds form an important habitat for microbes, sustaining a diverse array of both harmful and beneficial microbes (Nelson, 2004). Similar to the rhizosphere, the spermosphere is a region that surrounds seeds, and in which seed microbes, germinating seeds, and soil microbes may interact (Nelson, 2004). The microbiota living in this region, although usually short-lived as individual organisms, can have a persistent effect on seed germination and seedlings (Nelson, 2004; Delgado-Sánchez et al., 2011; Chen et al., 2012; Schiltz et al., 2015). Recently, research has revealed that microbes in the seed spermosphere and endosphere, which are less studied than other groups of symbionts, have the ability to promote seed germination and enhance plant growth during both abiotic and biotic stress (Truyens et al., 2015). For example, fungi isolated from *Opuntia* spp. (*Penicillium chrysogenum, Phoma* sp., and *Trichoderma koningii*) are involved in breaking seed dormancy and promoting germination (Delgado-Sánchez et al., 2011, 2013). Similarly, some seed-borne endophytic fungi from Ascomycota and Pleosporales have been reported to promote the growth and germination of *Phragmites australis* (Ernst et al., 2003). In addition, the effects of seed-associated microbiota on seed germination and plant growth are not limited to plant–fungal interactions; seed-associated bacteria have also been found to have similar functions in relation to plant fitness (Xu et al., 2014; Hardoim et al., 2015; Pitzschke, 2016). Therefore, it is reasonable to hypothesize that seed-associated microbes, including epiphytes and endophytes, play a more important role in modulating their host plant than previously thought.

High-throughput sequencing studies have identified a high frequency of *Cladosporium* spp. in seeds, specifically the inner seeds of a wide range of herbaceous plants (Ikeda et al., 2006; Lucero et al., 2011). Similarly, it has been reported that both endophytes and epiphytes associated with seeds play significant roles in seed germination and plant growth (Pitzschke, 2016; Tahtamouni et al., 2016). In rainy tropics, seed epiphytic fungi (*Penicillium* sp. and *Fusarium* sp.) have been shown to enhance seed germination (Tamura et al., 2008). Thus, exploration of these microbial communities using modern metagenomics has revealed there to be genetic and biochemical diversity in the spermosphere and endosphere of seeds.

## Plant growth promotion and stress tolerance

Although there has been a wide acceptance of the beneficial role of endophytes in plant growth and development, particularly in terms of their potential applications, seed-borne endophytes have been poorly explored. Beneficial seed-borne endophytes are thought to promote plant growth and mitigate stress (Truyens et al., 2015; Khamchatra et al., 2016; Shahzad et al., 2016, 2017c; Shearin et al., 2017); however, the underlying mechanisms remain largely unknown. The growth-promoting potential of seed-borne endophytes has been reported in many plants (Table 1). Several seed-borne bacterial and fungal endophytes produce compounds that either directly inhibit pathogen growth or indirectly strengthen plant resistance in defense against pathogenic attack (Bonos et al., 2005; Clarke et al., 2006; Tayung et al., 2012; Shahzad et al., 2017a). Yue et al. (2000) have determined the occurrence of numerous indole compounds, a sesquiterpene, and diacetamide from *Epichloë festucae*. Moreover, Shahzad et al. (2017a) report that the various organic acids produced by seed-borne endophytic *Bacillus amyloliquefaciens* acted to significantly inhibit the growth of pathogenic *Fusarium oxysporum in vitro*, and induced systemic resistance in tomato plants. Díaz Herrera et al. (2016) report the isolation from wheat seeds of the endophytes *Paenibacillus* sp., *Pantoea* sp., and *Bacillus* sp., which significantly enhanced plant growth and resistance against *F. graminearum*. Furthermore, *Epicholë* grass endophytes are also widely used in improving the survival and productivity of perennial ryegrass (Karpyn Esqueda et al., 2017). Turfgrasses infected with *E. festucae* showed a significantly improved resistance in comparison with non-inoculated turfgrasses against two of the main leaf spot pathogens, *Sclerotina homeocarpa* and *Laetisaria fuciformis* (Bonos et al., 2005; Clarke et al., 2006). However, it remains unclear whether this enhanced defense mechanism is attributable to metabolites produced by endophytes, secondary metabolites produced by plants in response to inoculation by endophytes, or competition between pathogenic microbes. Interestingly, in addition to their antagonistic capability against pathogenic microbes, seed endophytes also improve seed germination, mitigate abiotic stress, and enhance plant tolerance, features which are probably related to the ability of these microbes to produce secondary metabolites, siderophores, and ACC deaminase (Glick, 2012; Xu et al., 2014; Shahzad et al., 2017a,b). Moreover, the application of plant growth-promoting seed-borne bacterial endophytes may also facilitate the phyto- and bioremediation of contaminated soil. Mastretta et al. (2009) have shown in their study that, inoculation of tobacco plants with seed endophytes under Cd stress resulted in significantly improved plant growth, enhanced biomass, alleviation of Cd toxicity, and improved tolerance as compared to uninoculated plants. Truyens et al. (2015) also report enhanced phytoremediation of grasses following inoculation with seed-borne endophytes with the potential to solubilize phosphorus and produce indole-3-acetic acid (IAA), siderophores, ACC deaminase, and acetone. They also conclude that there are benefits to establishing Cd-tolerant seed-borne endophytes in Cd-contaminated areas during phytoextraction and phytostabilization; in non-exposed plants, endophyte inoculation considerably improved plant growth, whereas under conditions of Cd stress, inoculation augmented Cd uptake without disturbing plant growth. These results show that endophyte microbes such as these are promising in terms of applicability to phytoremediation.

## Future perspectives

Investigating the role of seed-borne, vertically transmitted bacterial and fungal endophytes opens new and exciting opportunities for applied research into plant–microbe interactions, given that these microbes can improve seed germination, promote seedling health, enhance plant growth, and mitigate stress. These abilities can be attributed to the production of extracellular enzymes, phytohormones, and secondary metabolites. Given the growth-promoting and biocontrol properties of these microbes, their potential applications as biofertilizers and in bioremediation should be supported.

It is presumed that an extensive proportion of the endophytic population in seeds has not yet been fully explored. Metagenomic studies will provide additional insight into seed endophyte populations, including the genera, their phenotypic attributes, and possible roles in both germination and plant advancement. Further research is required in order to investigate seed–endophyte interactions and their role in inducing defense resistance mechanisms against biotic at the molecular level and also to identify the genetic determinants involved in seed colonization, seed endophyte dispersal, and vertical transmission. Finally, exhaustive research is needed to determine the changes that occur in seed-associated endophytes during seed development, storage, and germination, in order to ensure a superior quality production of seeds.

## Author contributions

RS design the study. RS, AK, SB, and SA wrote the review manuscript. I-JL and AK critically reviewed the manuscript and supervised the manuscript drafting.

### Conflict of interest statement

The authors declare that the research was conducted in the absence of any commercial or financial relationships that could be construed as a potential conflict of interest.
